# Parasites in Cholera

**Published:** 1885-02

**Authors:** 


*Parasites in Cholera* (*Bricon*).—Pouchet and Vahl have found in choleraic discharges the *Vibrio Regula*, and this observation has been confirmed by Hassel, in 1873. During the same year, Pouchet, Brittan, Swayne and Budd described some *annular bodies*, *choleraic cells*, and choleraic fungi, classified by Busk and Williams in the *Credineæ*. Grove observed some *granular bodies* in the urine of cholerics and Klob found some *zooglea* colonies which developed in *leptothrix;* Thomé described a fungus which he named *cylindro-thœnium choleræ asiaticæ*. But most important and badly valued were the studies of Prof. Pacini.

Pacini, in the year 1854, noted in the fecal discharges of the cholerics, the presence of numerous bacilli, multiplied in the intestinal epithelium, causing its destruction. Bouchardat con. sidered the cholera as caused by a poison produced by *infusoria*, which were the cause of putrefaction in the swamps of Gauge. Haller attributed the cholera to the action of micrococci, which were nothing else but spores of the *Urocystis occulta*.

Gietl ascribed the disease to parasites. Lewis and Cunningham found in the blood of the cholerics masses of protoplasma, transparent and larger than the white corpuscles of blood, without nuclei or cellular membrane, having amœboid movements and minute granulations.

Danet attributed the cholera to a cryptogam greatly analogous to the *Oidium albicans*. He affirmed it to be identical to that described by Thomé, by Pacini and by Klebs.

Nedsoctski found bacteria in the dejections, in the vomited matter, in the urine, in the blood, etc., of choleric patients. Martin and Schweninger saw the uriniferous tubules completely obstructed by bacilli.

Hayen and Raynaud found in the fecal matter of cholerics numerous organisms without any special form. They observed bacteria, vibriones, and bacilli, but similar to those usually found in the fæces of sound or sick individuals, and in every epoch of life.

